# Epigenetic Mechanisms Underlying COVID-19 Pathogenesis

**DOI:** 10.3390/biomedicines9091142

**Published:** 2021-09-02

**Authors:** Syuzo Kaneko, Ken Takasawa, Ken Asada, Norio Shinkai, Amina Bolatkan, Masayoshi Yamada, Satoshi Takahashi, Hidenori Machino, Kazuma Kobayashi, Masaaki Komatsu, Ryuji Hamamoto

**Affiliations:** 1 Division of Medical AI Research and Development, National Cancer Center Research Institute, 5-1-1 Tsukiji, Chuo-ku, Tokyo 104-0045, Japan; sykaneko@ncc.go.jp (S.K.); ktakazaw@ncc.go.jp (K.T.); ken.asada@riken.jp (K.A.); norio.shinkai@riken.jp (N.S.); abolatka@ncc.go.jp (A.B.); masyamad@ncc.go.jp (M.Y.); satoshi.takahashi.fy@riken.jp (S.T.); hmachino@ncc.go.jp (H.M.); kazumkob@ncc.go.jp (K.K.); maskomat@ncc.go.jp (M.K.); 2 Cancer Translational Research Team, RIKEN Center for Advanced Intelligence Project, 1-4-1 Nihonbashi, Chuo-ku, Tokyo 103-0027, Japan; 3 Department of NCC Cancer Science, Graduate School of Medical and Dental Sciences, Tokyo Medical and Dental University, 1-5-45 Yushima, Bunkyo-ku, Tokyo 113-8510, Japan; 4 National Cancer Center Hospital, Department of Endoscopy, 5-1-1 Tsukiji, Chuo-ku, Tokyo 104-0045, Japan

**Keywords:** COVID-19, SARS-CoV-2, epigenetics, ACE2, DNA methylation, histone modifications, non-coding RNA

## Abstract

In 2019, a novel severe acute respiratory syndrome called coronavirus disease 2019 (COVID-19), caused by severe acute respiratory syndrome coronavirus 2 (SARS-CoV-2), was reported and was declared a pandemic by the World Health Organization (WHO) in March 2020. With the advancing development of COVID-19 vaccines and their administration globally, it is expected that COVID-19 will converge in the future; however, the situation remains unpredictable because of a series of reports regarding SARS-CoV-2 variants. Currently, there are still few specific effective treatments for COVID-19, as many unanswered questions remain regarding the pathogenic mechanism of COVID-19. Continued elucidation of COVID-19 pathogenic mechanisms is a matter of global importance. In this regard, recent reports have suggested that epigenetics plays an important role; for instance, the expression of angiotensin I converting enzyme 2 (ACE2) receptor, an important factor in human infection with SARS-CoV-2, is epigenetically regulated; further, DNA methylation status is reported to be unique to patients with COVID-19. In this review, we focus on epigenetic mechanisms to provide a new molecular framework for elucidating the pathogenesis of SARS-CoV-2 infection in humans and of COVID-19, along with the possibility of new diagnostic and therapeutic strategies.

## 1. Introduction

Coronavirus disease 2019 (COVID-19) is an infectious disease caused by severe acute respiratory syndrome coronavirus 2 (SARS-CoV-2). Its symptoms are varied and range from mild to severe; common symptoms include headache, loss of sense of smell and taste, nasal congestion and leakage, cough, myalgia, sore throat, fever, diarrhea, and difficulty breathing [1,2,3,4,5]. Of those with symptoms significant enough to be classified as patients in a study in China, most (81%) had mild to moderate symptoms (up to mild pneumonia), 14% had severe symptoms (dyspnea, hypoxia, or more than lung involvement on imaging), and 5% had severe symptoms (respiratory failure, shock, or multiple organ failure) [6]. COVID-19 spread around the world within a short time and was declared a pandemic by the World Health Organization (WHO) on 11 March 2020. Since the end of 2020, the emergence of SARS-CoV-2 variants with genetic mutations that may affect transmission, severity, and antigenicity has become a major problem. In particular, outbreaks of B.1.1.7 (Alpha variant), first detected in the UK, B.1.351 (Beta variant), first detected in South Africa, P.1 (Gamma variant), first detected in Japan among returnees from Brazil, and B.1.617.2 (Delta variant), first detected in India, are of global concern [7,8].

Under these circumstances, the development of vaccines aiming to provide acquired immunity to humans against SARS-CoV-2 has been progressing, and as of July 2021, multiple vaccines with varying methods and manufacturers have been developed, ranging from those that have already been inoculated to those under development (mRNA vaccines, DNA vaccines, virus vector vaccines, inactivated vaccines, recombinant protein vaccines, peptide vaccines, etc.) [9]. In particular, the results of a large placebo-controlled trial of vaccines from Pfizer and Moderna have shown that two repeated doses of the vaccine are highly effective in preventing COVID-19 (more than 90%) [10,11]. Based on the results of these clinical trials, vaccination with multiple types of COVID-19 vaccines developed by several pharmaceutical companies began worldwide at the end of 2020 or in early 2021 [12]. While hopes for pandemic containment through vaccination are increasing, the emergence of SARS-CoV-2 variants with amino acid mutations in the antigenic determinants of spike proteins, as described above, has been reported worldwide. Further, since there is a possibility that unknown SARS-CoV-2 variants may emerge in the future, it is necessary to continue with basic infection control measures without overconfidence in the effectiveness of vaccines.

Currently, drugs such as Remdesivir, Dexamethasone, Baricitinib and Heparin are used as treatments for COVID-19; however, there are still few specific and effective treatments for COVID-19 [13,14]. This is because there are still many unanswered questions regarding the pathogenic mechanism of COVID-19. Elucidating the pathogenic mechanism of COVID-19 is thus essential to establish effective treatment for patients with severe disease and to develop novel therapeutic agents. In this regard, epigenetics status such as histone modification of host cells is known to be altered upon infection with RNA viruses including coronaviruses, and the importance of epigenetics in the pathogenic mechanism of SARS-CoV-2 infection in humans and COVID-19 was recently pointed out.

Epigenetics is commonly defined as the study of heritable phenotypic changes without altering the DNA sequence. The Greek prefix epi- (ἐπι) in epigenetics implies a function “above” or “in addition to” the traditional genetic base [15]. Over the past two decades, epigenetic regulators have been implicated as critical factors in many pathways related to the development and progression of cancer and other diseases, including cell cycle regulation, invasiveness, signaling pathways, chemotherapy resistance, and immune evasion [16,17,18,19,20,21,22,23,24,25,26,27]. The three basic systems of epigenetic regulation are DNA methylation of gene regulatory regions; histone protein modifications such as methylation, acetylation, phosphorylation, and sumoylation; and non-coding RNAs [15]. Many techniques for epigenetics analysis have already been developed, and this field is steadily undergoing technological innovation [15,28,29]. In this review, we present the latest findings on the importance of epigenetics in the mechanisms of human infection with SARS-CoV-2 and pathogenesis of COVID-19, and discuss future diagnostic and therapeutic strategies for COVID-19 targeting epigenetics.

## 2. The Life Cycle of SARS-CoV-2

The SARS-CoV-2 genome is composed of single-stranded RNA [30]. SARS-CoV-2 shares 96% genome sequence identity with BatCoV RaTG13 [31]; 90% identity with Pangolin-CoVs [32]; 88% genome sequence identity with two bat-derived coronaviruses, bat-SL-CoVZC45 and bat-SL-CoVZXC21; 79% identity with SARS-CoV; and 50% with Middle East respiratory syndrome coronavirus [31,32]. Although SARS-CoV-2 has highly identical sequences with the above viruses, SARS-CoV-2 only has a functional furin cleavage site at the spike (S) protein [33,34].

The S protein expressed on the surface of the viral particles is essential to the initial steps of coronavirus infection. The S protein comprises the receptor-binding subunit S1 and the membrane-fusion subunit S2 [35,36,37]. The receptor binding S1 consists of two subdomains, an N-terminal domain and a C-terminal domain [38,39].

**Figure 1:**
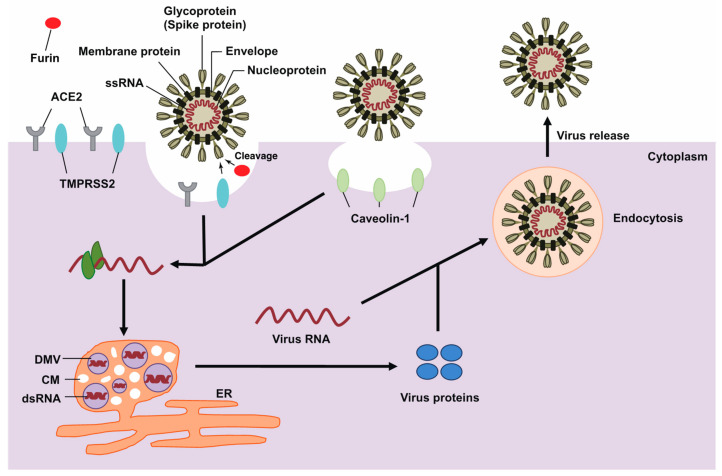
A schematic representation of the life cycle of coronavirus. Coronavirus spike protein (trimers) binds with membrane surface proteins of host cells to facilitate viral entry. Uncoated genomic RNA is then translated, and viral components are replicated. Viral proteins are assembled for maturation, and the newly packaged viral particles are released.

The viral S protein binds to angiotensin I converting enzyme 2 (ACE2) as entry receptor. In addition, the S protein of SARS-CoV-2 cleaves by transmembrane serine protease-2 (TMPRSS2) and furin protease, key factors in its host cell entry [37,40,41]. Interestingly, studies have attempted to explain the global pandemic of COVID-19 based on hardness and phylogenetic analysis of the outer shell (M protein) of SARS-CoV-2, and there may be other factors besides the S protein of the virus that characterize SARS-CoV-2 [42]. SARS-CoV-2 is hypothesized to use clathrin-dependent endocytosis [43,44,45], the most common endosomal pathway, to facilitate viral entry. Caveolae-dependent uptake, which is the other pathway, is considered controversial regarding coronavirus entry into host cells and is potentially dependent on cell type [46,47]. Following genome release into the cytosol, viral genomic RNA is uncoated and translated for the synthesis of non-structural viral proteins. Importantly, this intrinsic disorganization of viral proteins is an inherent feature and a strategy of viruses to disrupt host nucleocytoplasmic transport to benefit their own replication [48]. Viral RNA is then replicated and subgenomic RNAs are translated in double-membrane vesicles, as previously reported in several viruses, including coronavirus [49,50,51,52]. Multiple components, such as host membrane-derived double-membrane spherules, convoluted membranes, and the endoplasmic reticulum (ER), are used to protect and support the transcribed genomic RNA and are hallmarks of viral infections. Translated structural proteins then translocate into the ER and transit through the ER-to-Golgi intermediate compartment, wherein N-encapsidated, newly produced genomic RNA is packaged in the lumen of secretory vesicles [53,54]. Viruses are then secreted from the infected cells by exocytosis, and the aforementioned process is repeated in new cells (Figure 1). Notably, D614G substitution in the receptor-binding domain of the S protein of SARS-CoV-2 results in the change of a single amino acid, which enhances binding affinity to the ACE2 receptor and increases viral entry into host cells [55]. Thus, to enhance host cell defenses, it is important to understand how viral RNA infects host cells.

## 3. RNA Modifications of the SARS-CoV-2 Genome

RNA editing induces substitution in the viral genome and can potentially affect viral infection. RNA epigenetics can be divided into two main categories according to the mechanisms involved. One occurs when RNA modifications are catalyzed by methyltransferases (known as “writer proteins”) such as METTL3, and the other occurs when RNA editing is catalyzed by editing enzymes. RNA editing by host deaminases is an innate restriction process used as a defense mechanism against viral infection [56]. RNA sequencing of SARS-CoV-2 isolated from the bronchoalveolar lavage fluid of patients with COVID-19 revealed instances of RNA editing, such as A to G and U to C substitutions, and was accompanied by evidence of adenine to inosine (A-to-I) reactions catalyzed by RNA-specific adenosine deaminase (ADAR). In addition, restriction of viral replication was observed with C to U and G to A substitutions by apolipoprotein B mRNA editing enzyme catalytic polypeptide [57].

Furthermore, using RNA antisense purification and quantitative mass spectrometry in SARS-CoV-2-infected human cells, ADAR was found to interact with SARS-CoV-2 RNA [58]. The authors identified 699 proteins, of which 583 were detected with multiple peptides using liquid chromatography with tandem mass spectrometry. Viruses are known to utilize the host RNA editing machinery, and the resulting substitutions can lead to both proviral and antiviral effects. Recent studies have described the distribution of RNA substitution in the SARS-CoV-2 genome, and have provided insights into the random mutation and the specific substitutions induced by editing enzymes [59,60].

## 4. *ACE2* Gene Expression

As ACE2 is an essential intracellular receptor that binds to the S protein encoded by SARS-CoV-2 to cause an infection, understanding the status and mechanism of *ACE2* gene expression is essential. Generally, *ACE2* is expressed in various human organs, and its organ- and cell-specific expression suggests that it is involved in regulating cardiovascular and renal function and fertility [61]. Interestingly, single-cell RNA sequence datasets revealed that ACE2 was coexpressed with TMPRSS2 within lung type II pneumocytes, ileal absorptive enterocytes, and nasal goblet secretory cells [62]. It is also known that young children exhibit lower *ACE2* expression compared to the adults [63]; the low risk of infection in children may thus be attributed to this age-dependent ACE2 expression [64].

Studies using biochemical approaches have examined the binding of transcription factors to DNA sequences upstream of the transcription start site (TSS) of *ACE2*. Using the human embryonic kidney cell line HEK293, it has been suggested that there is a functional hepatocyte nuclear factor 1β (HNF1β) binding site in the promoter region and HNF1β in turn promotes *ACE2* transcription [65]. Furthermore, HNF1α and HNF1β, which bind to three HNF1-binding motifs that are highly conserved among mammalian species, cooperatively regulate ACE2 activity in insulinoma cells [66]. The *ACE2* gene is known to produce various transcripts that are stimulated by interferons and has several different TSSs, wherein the selection of TSSs varies from organ to organ [67,68,69].

As the *ACE2* gene is located on the X chromosome, a gene dosage effect potentially regulates gene expression. However, new evidence indicates that COVID-19 is a gender-biased disease influenced by myriad variables ranging from biological to social factors, and it is thus difficult to determine the relationship between SARS-CoV-2 infection and sex [70].

## 5. Effect of DNA Methylation on *ACE2* Expression

**Figure 2:**
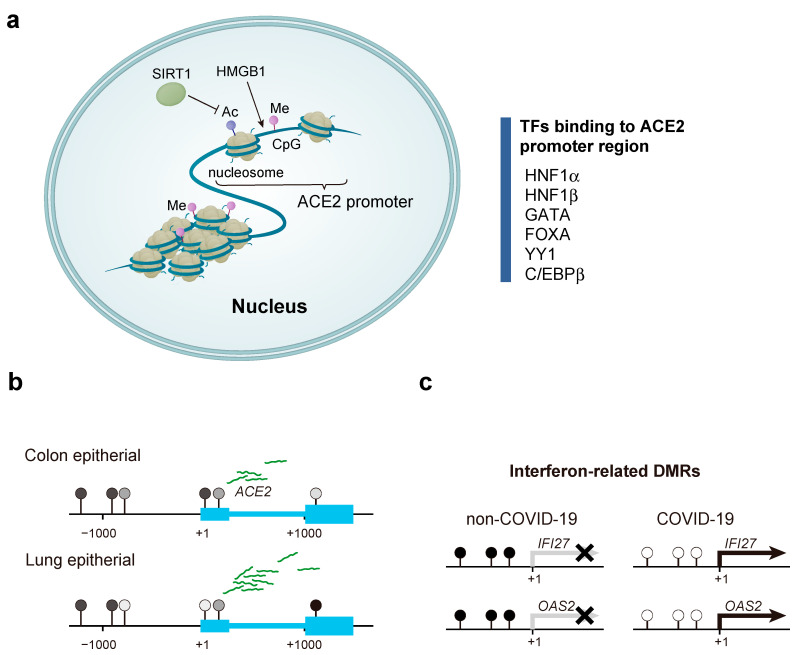
The effect of chromatin organization and DNA methylation on angiotensin I converting enzyme 2 (ACE2) gene expression in patients with coronavirus disease 2019 (COVID-19). (**a**) *ACE2* gene expression is subject to a variety of epigenetic controls. Here, we focus on transcription factors that bind to the ACE2 promoter region, factors that convert chromatin structure (HMGB1), histone deacetylases (SIRT1), and DNA methylation. (**b**) In cells with low *ACE2* expression, such as intestinal epithelial cells and vascular endothelial cells, CpG sites around the promoter are hypermethylated. On the contrary, in lung epithelial cells with high *ACE2* expression, the CpG sites surrounding the promoter are hypomethylated, suggesting that DNA methylation regulates *ACE2* gene expression. (**c**) Differentially methylated regions (DMRs), located around the promoters of interferon-related genes, are hypomethylated in patients with COVID-19 and are hypermethylated in patients without COVID-19. Balnis et al. performed a quantitative real-time polymerase chain reaction to estimate the expression of *IFI27* and *OAS2* and found that their expression was high in patients with COVID-19 and low in patients without COVID-19. This suggests that the interferon-related genes DMRs are hypomethylated in patients with COVID-19, resulting in the activation of these genes and pathways.

DNA methylation is an epigenetic modification reported to be associated with various clinical conditions, such as cancer and asthma [71,72,73]. The level of DNA methylation around promoters is thought to regulate the expression of proximal genes by altering the affinity of transcription factor binding. As mentioned above, Pedersen et al. performed promoter deletion analysis for *ACE2* and identified two regulatory promoter regions, the distal promoter region and proximal promoter region at −1509 bp to −928 bp and −454 bp to −1 bp from the TSS, respectively [66]. These promoter regions contain several transcription factor binding motifs such as HNF1α, HNF1β, GATA, FOXA, YY1, and C/EBPβ [66,74,75,76] (Figure 2a). The binding motif of C/EBPβ contains a CpG site, and the hypomethylation of the C/EBPβ binding motif is reported to induce angiotensinogen expression [77]. Therefore, DNA methylation status around the *ACE2* promoter may be involved in regulating *ACE2* gene expression. Comparative analysis of DNA methylation in various tissues has revealed that several CpG sites are hypomethylated in lung epithelial cells, which exhibit high ACE2 expression [78] (Figure 2b). Further, Cardenas et al. reported multiple methylation sites in the vicinity of the TSS and ACE2 gene body with variable degrees of methylation dependent on sex and race and a higher level of hypomethylation in females compared to that in males [79]. Although the platform used for the analysis was from a previous generation, Yang et al. reported that patients with asthma showed differentially methylated CpG sites in similar locations [80]. Wang et al. systematically analyzed the aberrant expression of ACE2 and TMPRSS2 in multiple human cancers and found that colorectal cancers with elevated gene expression had reduced DNA methylation levels. Since cancer is considered a risk factor for COVID-19, the outbreak of COVID-19 may require additional care, especially for colorectal cancer patients [81].

## 6. Alteration of DNA Methylation in Patients with COVID-19

The DNA methylation status in the host is reported to change with bacterial and viral infections [82], and analysis of the DNA methylation state is expected to aid in estimating the infection history and future disease severity [83]. Balnis et al. performed a comprehensive DNA methylation analysis using blood from hospitalized patients (COVID-19 and non-COVID-19) and healthy individuals [84] (Figure 2c) and found that although global DNA methylation levels did not differ between healthy and hospitalized individuals, 1089 hypo-differentially methylated regions (DMRs) and 416 hyper DMRs were found [84]. In addition, a comparison between patients with and without COVID-19 revealed 47 COVID-19 patient-specific DMRs, 36 of which were inversely correlated with the expression of neighboring genes. Moreover, gene ontology analysis of these 36 DMRs showed that ontologies involving defense response to the virus (27/36 DMRs) and interferon signaling (19/36 DMRs) were enriched [84]. Furthermore, 77 DMRs were identified by comparing patients with mild and severe COVID-19. The results of hierarchical cluster analysis of these 77 DMRs were consistent with the severity assessment indicated by the COVID-GRAM score [85], indicating that these DMRs can potentially act as biomarkers [84].

## 7. Histone Modifications Related to *ACE2* Gene Expression and COVID-19

Histone modifications are involved in human health conditions, aging, neurological diseases, and cancer development, which have been described in the great reviews [86,87,88]. An association between *ACE2* gene expression and histone modifications was reported previously [89]. Although core histones in nucleosomes are tightly arranged, histone-modifying enzymes can modify their tails. The counteracting action of various enzymes results in reversible epigenetic modifications. As described in other reviews, the action of enzymes, such as histone acetyltransferases, are balanced by those of histone deacetylases and the action of histone methyltransferases are countered by the action of histone demethylase, to maintain the epigenome [90,91]. Silent information regulator T1 (SIRT1), a histone deacetylase, is reported to be involved in the transcriptional regulation of *ACE2* expression [92], and this role is thought to be related to the protective role of SIRT1 against cellular stress. Therefore, nonsteroidal anti-inflammatory drugs that inhibit SIRT1 deacetylase activity are expected to exert unexpected anti-infective effects [93]. Notably, administration of atorvastatin to rabbits on a high cholesterol diet has been reported to upregulate *ACE2* expression via tissue-specific and promoter-specific histone modifications compared to that in their corresponding controls [94]. Importantly, the elevated *ACE2* expression in the lungs of patients with severe COVID-19-related complications may be due to histone modifications of several genes such as *HAT1*, *HDAC2*, and lysine demethylase 5 B (KDM5B) [89]. In particular, KDM5B affects chromatin accessibility by removing activated chromatin marks such as the dimethylation and trimethylation of histone H3 (H3K4) from lysine 4, thereby contributing to transcriptional regulation and DNA repair [95]. Inhibition of KDM5B in breast cancer cells has been shown to induce an interferon response, making the cells less susceptible to DNA and RNA viral infection [96]. Therefore, KDM5 demethylase is expected to be a potential target for COVID-19 prevention. Furthermore, diarylheptanoids, also known as diphenylheptanoids have been reported to induce epigenetic silencing of the *ACE2* gene mediated by HMGB1 [97,98,99,100,101], and this has attracted attention as a possible way to prevent COVID-19 infection. Intriguingly, it has been reported that the expression of TMPRSS2 and ACE2 was decreased by therapies directly targeting androgen receptor (AR) and inhibitors of bromodomain and extra terminal domain (BET) proteins, which are known epigenetic regulators of AR transcriptional activity. Furthermore, these treatments reduced SARS-CoV-2 infection in a cellular model. Therefore, these findings support further research on AR and BET inhibitors as potential treatments for COVID-19 [102,103].

**Figure 3:**
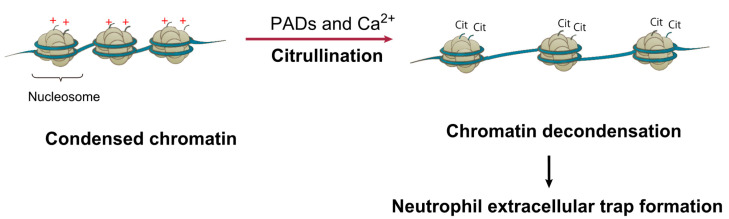
Citrullination of histone tails via peptidylarginine deiminase (PAD) leads to chromatin decondensation. PAD-mediated histone tail citrullination of positively charged arginine leads to generation of uncharged citrullines and, subsequently, chromatin decondensation. Wang et al. proposed that histone hypercitrullination mediates chromatin decondensation and neutrophil extracellular trap formation (ref. [106]).

Histone modifications are not only involved in the transcriptional regulation of ACE2, but also have important pathophysiological functions in COVID-19. Elevated citrullination of histone H3 (Cit-H3) in the serum of COVID-19 patients was recently reported [104]. Citrullination (deamination) of arginine residues in histones leads to generation of uncharged citrullines, facilitates chromatin decondensation, and enhances the accessibility of chromatin [105,106,107] (Figure 3). Histone citrullination is catalyzed by the enzyme peptidylarginine deiminase, a family of calcium-dependent enzymes that regulate immune activity and are involved in neutrophil extracellular traps (NETs) [106,107]. Pathologically, NETs are a biological defense response of neutrophils, which are triggered by infection; cell death via NETosis, which is different from cell necrosis and apoptosis, results in the release of neutrophil DNA into the extracellular space to form net-like structures [108]. As there is a positive correlation between Cit-H3 levels and platelet counts, Cit-H3 may contribute to abnormal platelet counts. Furthermore, a recent report suggested that NETs could be markers of COVID-19 severity [104]. Therefore, in the future it will be imperative to investigate the prognostic impact of histone Cit-H3 conversion on SARS-CoV-2 infection and venous thrombosis from both molecular biological and clinical perspectives.

## 8. *ACE2* Gene Regulation Mediated by miRNAs, lncRNAs, and circRNAs

Recently, the number of reports regarding the role of microRNAs (miRNAs) in regulating the *ACE2* gene has been increasing. miRNAs are non-coding, single-stranded, small RNAs that function by post-transcriptionally inhibiting the expression of target genes in various organisms, from viruses to higher eukaryotes [109,110,111,112,113,114]. These small RNAs function by complementarily binding to the 3′-untranslated region (UTR) and sometimes the 5′-UTR or coding regions of their target mRNAs. miRNAs are involved in numerous disease and physiological functions, such as cancer [115], degenerative neuro disorders [116], cardiovascular disease [117], and immunity [118]. miRNAs are also often identified as biomarkers of viral infections, and their contribution to host–pathogen interactions during viral infections is being investigated [119,120,121]. Recently, *ACE2* mRNA and ACE2 protein levels were reported to be repressed by miR-200c in primary rat cardiomyocytes and human-induced pluripotent stem cell-derived cardiomyocytes, suggesting a direct link between miR-200c and the regulation of *ACE2* expression [122]. Many other miRNAs are thought to have the potential to regulate *ACE2*, but most of them have not been carefully investigated [123,124]. Furthermore, it has been suggested that TMPRSS2 transcription is regulated by miR-98-5p in two human endothelial cell types, derived from the lung and from the umbilical vein, suggesting strict control by the miRNA-target network [125].

Long noncoding RNAs (lncRNAs) and circular RNAs (circRNAs) have also been implicated in SARS-CoV-2 infection [126,127,128]. Like miRNAs, lncRNAs and circRNAs are a class of non-coding RNAs, but lncRNAs are typically 1000–10,000 residues in length, and circRNAs are circular RNAs processed by back splicing [129,130]. Comparison analysis between normal human bronchial epithelial cells and SARS-CoV-2 infected cells identified several differentially expressed lncRNAs, and the differences of the expression were also confirmed among COVID-19 patients [126]. Involvement of circRNAs in several viral infections such as hepatitis B virus and human papillomavirus were recently reported [128]. For infection of SARS-CoV-2, the two circRNAs, Ppp1r10 and C330019G07RiK, were reported as a part of quintuple competing endogenous RNA networks consisting of miRNA, lncRNA, circRNAs, mRNA, and transcription factor by in silico study [127].

## 9. Identification of SARS-CoV-2 Infection Factors Using Genome-Wide Screening Analysis

The cutting-edge technologies such as next-generation sequencing (NGS) and CRISPR systems have been contributing to the finding of new insights and development of new diagnostic methods for COVID-19 [131,132,133]. The studies using NGS revealed the whole sequences of SARS-CoV-2 at just 4 months from the COVID-19 pandemic [134], and the sequences were beneficial for understanding the virus genomic variants and pathogenetic mechanisms [131,132,133]. These innovative technologies are useful for understanding not only the virological features of SARS-CoV-2 but also the response of patients in a molecular biological aspect. A series of reports have identified factors associated with SARS-CoV-2 infection using genome-wide screening analysis [97,135,136]. Wei et al. identified epigenetic regulatory genes involved in diverse biological processes, such as chromatin remodeling, histone modification, intracellular signaling, and RNA regulation, as candidate host genes that affect SARS-CoV-2 infection. For example, HMGB1 appears to play a novel role in epigenetically regulating ACE2 expression, thus increasing susceptibility to SARS-CoV-2 infection (Figure 2a). HMGB1 encodes a non-histone nuclear DNA-binding protein, belonging to the high mobility group-box superfamily, that regulates transcription and is involved in DNA organization [137,138]. This protein is also known to act as a danger-associated molecular pattern molecule that amplifies immune responses during tissue injury [139]. Notably, HMGB1 regulates *ACE2* expression in a cell-intrinsic manner rather than functioning as a cytokine or alarmin, suggesting a precise mechanism for HMGB1 involvement in SARS-CoV-2 infection [97].

Furthermore, the gene encoding the SWI/SNF chromatin remodeling complex was identified as a SARS-CoV-2 provirus, demonstrating the importance of this complex as a pathogen [97]. Previously, the SWI/SNF complex has been reported to be composed of the ATPase subunits SMARCA2 or SMARCA4, and it catalyzes non-catalytic scaffold core expression via ARID1A, which has no inherent DNA sequence specificity [140,141]. Its target specificity is thought to be conferred by the recruitment of DNA-binding proteins to target sites on the genome and by sliding nucleosomes to regulate chromatin accessibility and gene expression.

However, TMPRSS2, TMPRSS4, and FURIN [142,143], which are considered proviral genes for SARS-CoV-2 infection, were not identified in this screening. This discrepancy may be due to technical difficulties, such as the use of CRISPR libraries and variation in gRNA expression levels in the cell type used.

## 10. Discussion

In this review, we presented the latest findings on the mechanisms of SARS-CoV-2 infection and COVID-19 pathogenesis with a focus on epigenetics. Since the discovery of the double helix structure of DNA by J.D. Watson and F.H. Crick in 1953 [144], molecular biology has emerged and various life mechanisms have been elucidated at the genetic level. In addition, with the completion of the Human Genome Project in 2003 and the elucidation of the human whole genome, there has been a growing momentum for the application of genomic information to medicine, known as genomic medicine [15,27,28]. U.S. President Barack Obama announced the Precision Medicine Initiative in 2015, leading to a worldwide push for precision medicine, wherein patients are selected for optimal treatment based on genomic mutations [145].

On the contrary, since the latter half of the 20th century, the importance of changes in gene expression or cellular phenotype that are inherited after cell division without changes in DNA sequence has been indicated, and a new research field called epigenetics has developed [15]. In fact, when elucidating the mechanisms of diseases such as cancer, it is difficult to determine the entire pathogenesis based on genetic mutations alone, and the importance of conducting epigenetics research at the same time has been recognized worldwide. Further, considering the ongoing promotion of precision medicine, it is difficult to adequately screen patients based on genetic mutations alone, and epigenetic information must also be used appropriately. In clinical practice, DNA methylation is used for diagnosis [146], and HDAC inhibitors and DNA methylation inhibitors are used as therapeutic agents [147,148]. Furthermore, clinical research and clinical trials of new drug types, such as histone methyltransferase inhibitors and histone demethylase inhibitors, are currently underway worldwide for clinical application [149,150,151,152]. Considering these developments, it is important to conduct research focusing on epigenetics to elucidate pathological mechanisms and to develop new diagnostic and therapeutic methods for COVID-19. In this article, we also introduced the observation of DMRs unique to patients with COVID-19, which may serve as biomarkers for predicting the disease severity and the possibility of functioning as a novel therapeutic agent by epigenetically regulating *ACE2* expression. Since the involvement of epigenetics factors such as histone deacetylase in *ACE2* expression has been indicated, there is a possibility of more effective treatment for COVID-19, for example, by combining HDAC inhibitors, which are already approved by the FDA, with Remdesivir or Dexamethasone.

Since COVID-19 was first reported in December 2019, it has only been one year and eight months, and many unanswered questions remain regarding its pathological mechanisms. It is hoped that COVID-19 will be controlled as vaccination progresses, but with SARS-CoV-2 variants being reported consecutively, the situation remains unpredictable. We thus believe that it is of global importance to continue elucidating the pathological mechanisms of COVID-19 and to develop new diagnostic methods and therapeutic strategies to free people worldwide from the threat of COVID-19 and to enable them to lead safe and secure daily lives. Epigenetics is also an important subject of research, and we hope that the clinical importance of epigenetics in the pathogenesis of COVID-19 will be further clarified in the future by conducting analyses using larger clinical samples.

## Data Availability

Not applicable.
